# A Comparative Evaluation of Three Different Attachment Methods for a Video Laryngoscope in a Surgical Setting

**DOI:** 10.1002/lio2.70172

**Published:** 2025-06-02

**Authors:** Linus L. Kienle, Leon R. Schild, Andreas M. Seitz, Viola D. Hahn, Jens Greve, Thomas K. Hoffmann, Patrick J. Schuler, Felix Boehm

**Affiliations:** ^1^ Department of Otorhinolaryngology, Head and Neck Surgery Ulm University Medical Centre Ulm Germany; ^2^ Surgical Oncology Ulm i2SOUL Consortium Ulm Germany; ^3^ Institute of Orthopaedic Research and Biomechanics Ulm University Medical Centre Ulm Germany; ^4^ Department of Otorhinolaryngology Heidelberg University Medical Centre Heidelberg Germany

**Keywords:** difficult laryngeal exposure, force measurement, laryngeal surgery, TLM, video laryngoscopy

## Abstract

**Introduction:**

The prerequisite for transoral microsurgery of laryngeal lesions is an uninterrupted line of sight to the operative field. Patients with cervical stiffness or anatomical variations that prevent adequate laryngeal exposure are typically unsuitable for this procedure. In such cases, a curved video laryngoscope may facilitate improved access to the larynx. However, conventional suspension laryngoscopy setups are incompatible with standard video laryngoscopes.

**Objective:**

This study evaluates three attachment methods for integrating a curved video laryngoscope into a surgical setup, focusing on their resistance to external forces.

**Methods:**

This study assessed three different attachment methods (3D‐printed clamp vs. metal bracket vs. articulated stand) for a video laryngoscope in a surgical setup. External forces, both lateral and rotational (torque), were applied and continuously measured until laryngeal visualization was compromised by displacement of the video laryngoscope.

**Results:**

The metal bracket demonstrated significantly (*p* < 0.001) higher resistance to lateral forces (median 184.49 N, 95% CI [181.59–189.61 N]) compared to the articulated stand (median 88.16 N, 95% CI [76.73–88.98 N]) and the 3D‐printed clamp (median 55.59 N, 95% CI [54.74–57.58 N]). The articulated stand exhibited significantly (*p* < 0.005) greater torque resistance (median 9.57 N m, 95% CI [5.65–9.87 N m]) compared to the metal bracket (median 1.58 N m, 95% CI [1.57–2.13 N m]) and the 3D‐printed clamp (median 2.46 N m, 95% CI [2.24–2.79 N m]).

**Conclusion:**

Overall, the articulated stand outperformed the other attachment methods, displaying robust resistance to lateral forces and superior rotational stability.

**Level of Evidence:**

Level 4.

## Graphical abstract

The study presents a systematic evaluation of innovative attachment methods for integrating a curved video laryngoscope into surgical setups, addressing a significant clinical need for improving laryngeal exposure in patients with complex anatomical or mobility challenges. In this work, we assessed the performance of three attachment systems—3D‐printed clamp, metal bracket, and articulated stand—under conditions simulating external surgical forces.

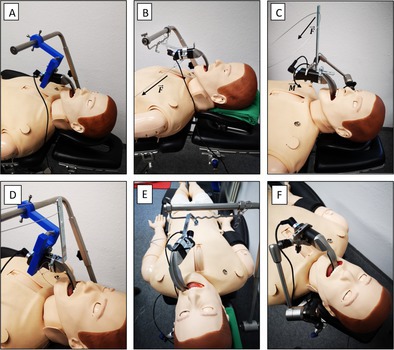

## 1 Introduction

Laryngeal cancer ranks among the most common neoplasms of the head and neck region. Early‐stage disease can often be successfully treated using transoral laser microsurgery (TLM). The procedure has been established over decades and is associated with superior functional results as well as good 5‐year survival rates [1]. The fundamental requirements for TLM are adequate exposure of laryngeal structures and an uninterrupted line of sight to the tumor site, facilitated by a rigid operating laryngoscope. However, exposure of the target region using an operating laryngoscope can be challenging due to a complex anatomy or cervical spine stiffness [2, 3]. As a result, these patients may require more invasive open surgery, which is associated with longer recovery time, increased morbidity, and usually a worse functional outcome [4, 5]. The alternative treatment option is radiotherapy, which is often less preferred by patients due to its prolonged treatment duration and associated side effects.

Curved video laryngoscopes have been employed in anesthesia for several years to enhance visualization of the larynx. These systems typically provide effective visualization of the larynx for consecutive tracheal intubation even in patients with known or predicted difficult airways [6]. Multiple studies have investigated the application of video laryngoscopy for transoral head and neck surgery. Case studies have demonstrated successful utilization of a curved video laryngoscope for diagnostic and therapeutic interventions of the larynx [7, 8, 9], hypopharynx [8, 9], and base of the tongue [8, 10]. These investigations established video laryngoscopy as a safe and effective alternative when conventional direct laryngoscopy provided insufficient exposure of the operative field.

Our research group developed a novel prototype system for laryngeal surgery based on a curved video laryngoscope equipped with two working channels for the use of flexible instruments [11]. In conventional direct laryngoscopy, a pillar is attached to the operating laryngoscope, which is placed on the patient's chest using a pillow or special support rod and provides stable visualization of the operating field. However, this attachment method is incompatible with standard video laryngoscope designs. Consequently, new attachment methods for surgical systems that use a curved video laryngoscope for visualization of the laryngeal operating field need to be developed and evaluated. The objective of this study is to determine the most effective attachment methods for securely performing laryngeal surgery using a curved video laryngoscope.

## 2 Materials and Methods

### 2.1 Video Laryngoscope

**FIGURE 1:**
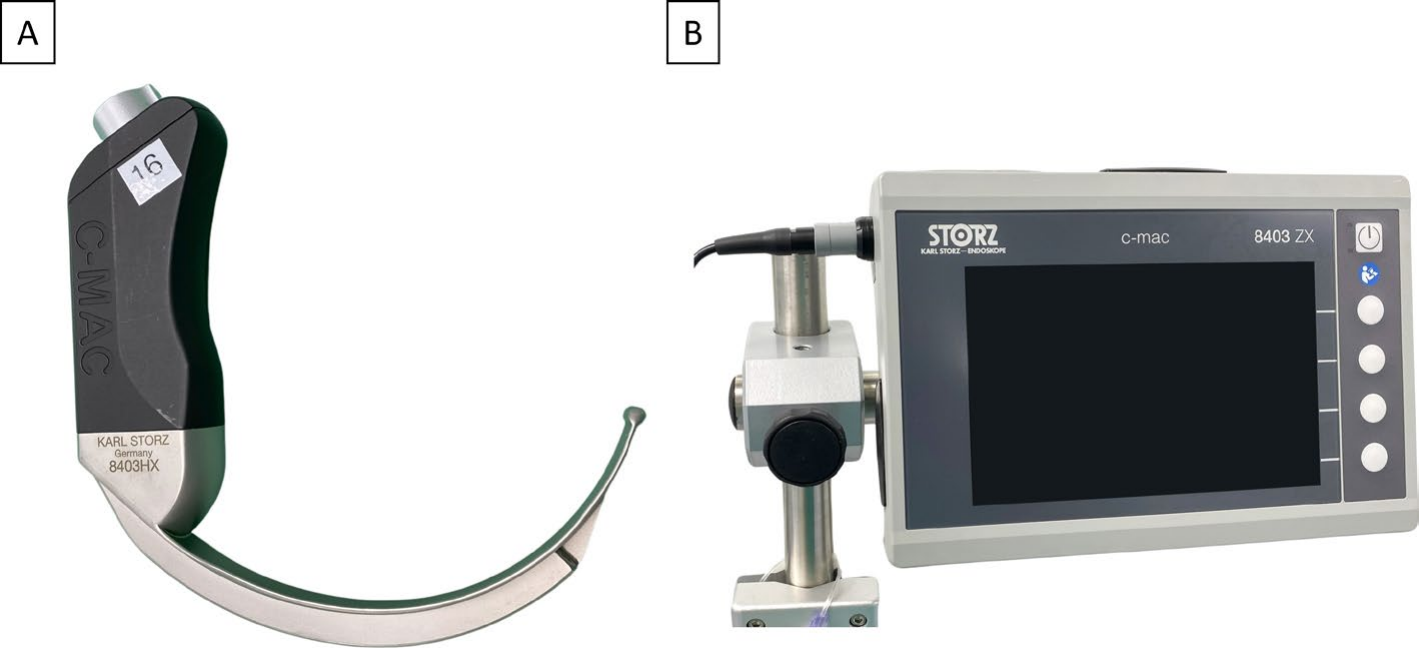
(A) Video‐laryngoscope with hyperangulated blade (D‐Blade) (B) 7‐in. monitor (C‐MAC, Karl Storz, Tuttlingen, Germany).

For the experiments, a video laryngoscope (C‐MAC, Karl Storz, Tuttlingen, Germany) with a hyperangulated blade (D‐Blade), particularly suited for difficult airways, was employed (Figure 1A). The video laryngoscope's image was displayed on a 7‐in. monitor (8403 ZX, Karl Storz) with a resolution of 1200 × 800 pixels (Figure 1B). The technical application of the surgical prototype on an intubation dummy as well as a body donor has been previously documented by Schild et al. [11].

### 2.2 Attachment Methods

Three different attachment methods for the video laryngoscope were evaluated.

#### 2.2.1 3D‐Printed Clamp

**FIGURE 2:**
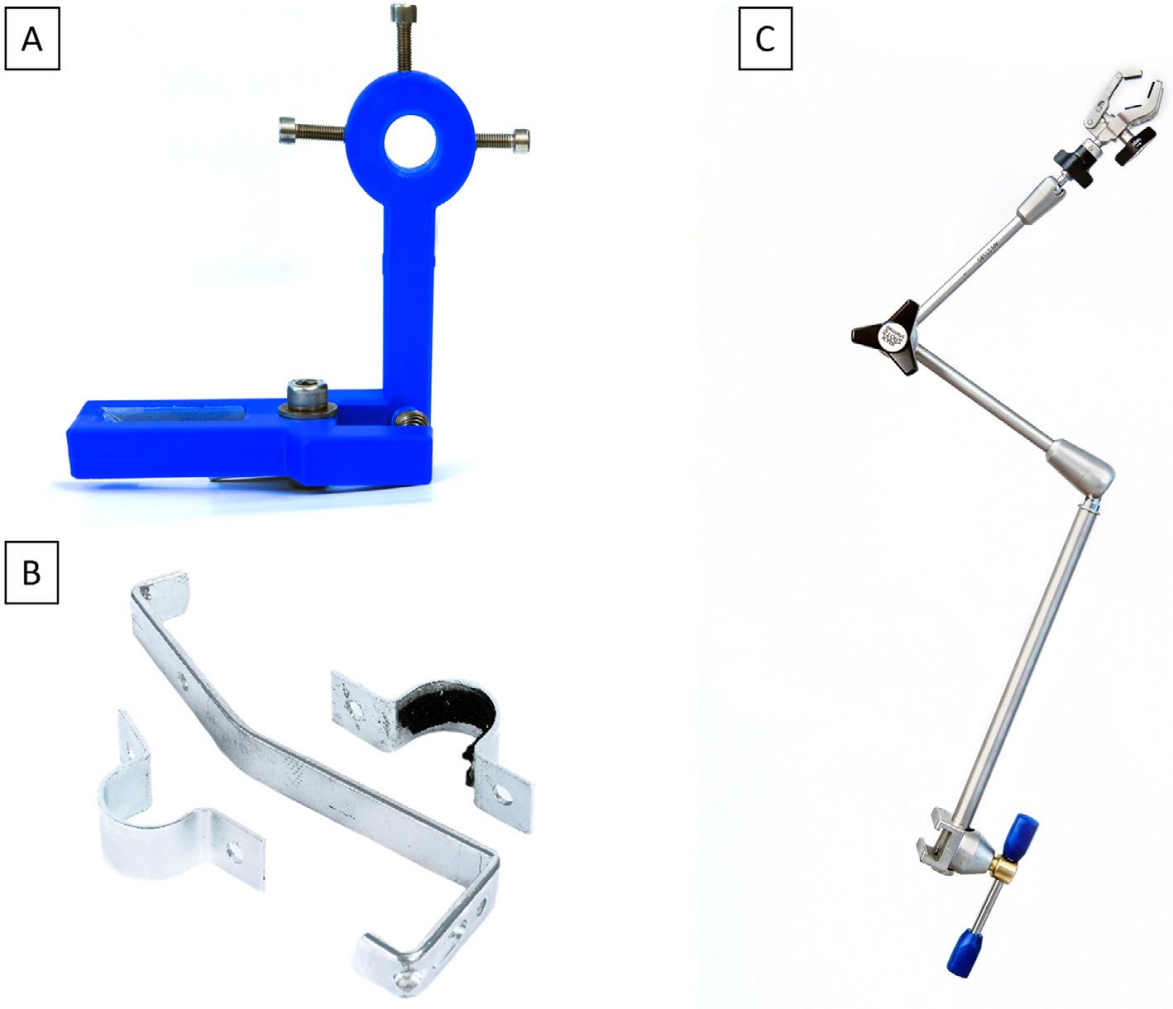
Different attachment methods for the surgical prototype evaluated in the study. (A) 3D‐printed spring‐loaded clamp. (B) Metal bracket. (C) Articulated stand.

The L‐shaped clamp is designed using CAD software (AutoCAD, Autodesk, San Rafael, USA) and fabricated from PLA using a 3D printer (i3 Mega, Anycubic, Shenzhen, China). The vertical leg of the L‐shape features a circular opening at the top, which allows attachment to an operating table crossbar via three set screws. The horizontal leg of the L‐shape is designed to secure the video laryngoscope and includes two clamping jaws with a rectangular slot to accommodate the handle of the video laryngoscope. The jaws, which enclose the handle of the video laryngoscope from both sides, are compressed by a 6 mm steel spring to hold the video laryngoscope securely in place. The 3D‐printed spring‐loaded clamp developed by the authors is illustrated in Figure 2A. The CAD files for the 3D‐printed clamp are available upon request from the authors and will be provided free of charge for scientific purposes.

#### 2.2.2 Metal Bracket

The metal bracket, designed by the authors, is constructed from an S‐shaped stainless‐steel rail and a circular bracket. The video laryngoscope handle is enclosed by the circular bracket, which is secured with a screw on each side. The interior of the circular bracket is lined with rubber to ensure a secure and precise fit around the laryngoscope handle. The curved shape of the stainless‐steel rail allows it to be connected to an operating table crossbar. The components of the metal bracket construction are depicted in Figure 2B.

#### 2.2.3 Articulated Stand

The articulated stand (28272HA, Karl Storz, Tuttlingen, Germany), illustrated in Figure 2C, is made from stainless steel and features a central mechanical clamp that simultaneously locks all three joints, allowing for versatile positioning of the video laryngoscope across a broad range of motion. The stand measures 30 cm in height and 67 cm in total length with an operational range of 37 cm. The stand can be attached to the siderails of an operating table via a rotation socket. The video laryngoscope is connected to the articulated stand using a size‐adjustable clamping jaw (28272UFN, Karl Storz, Tuttlingen, Germany), which grips the handle from two sides.

### 2.3 Measurement Equipment

The measurement was performed with a force sensor (KD40s, ME‐Meßsysteme, Henningsdorf, Germany) rated for 5kN. The sensor signal was amplified using an eight‐channel digital measuring amplifier (GSV‐8DS, ME‐Meßsysteme GmbH, Henningsdorf, Germany), sampled at a rate of 10 Hz, and transferred to a computer via a USB connection.

### 2.4 Experimental Setup

**FIGURE 3:**
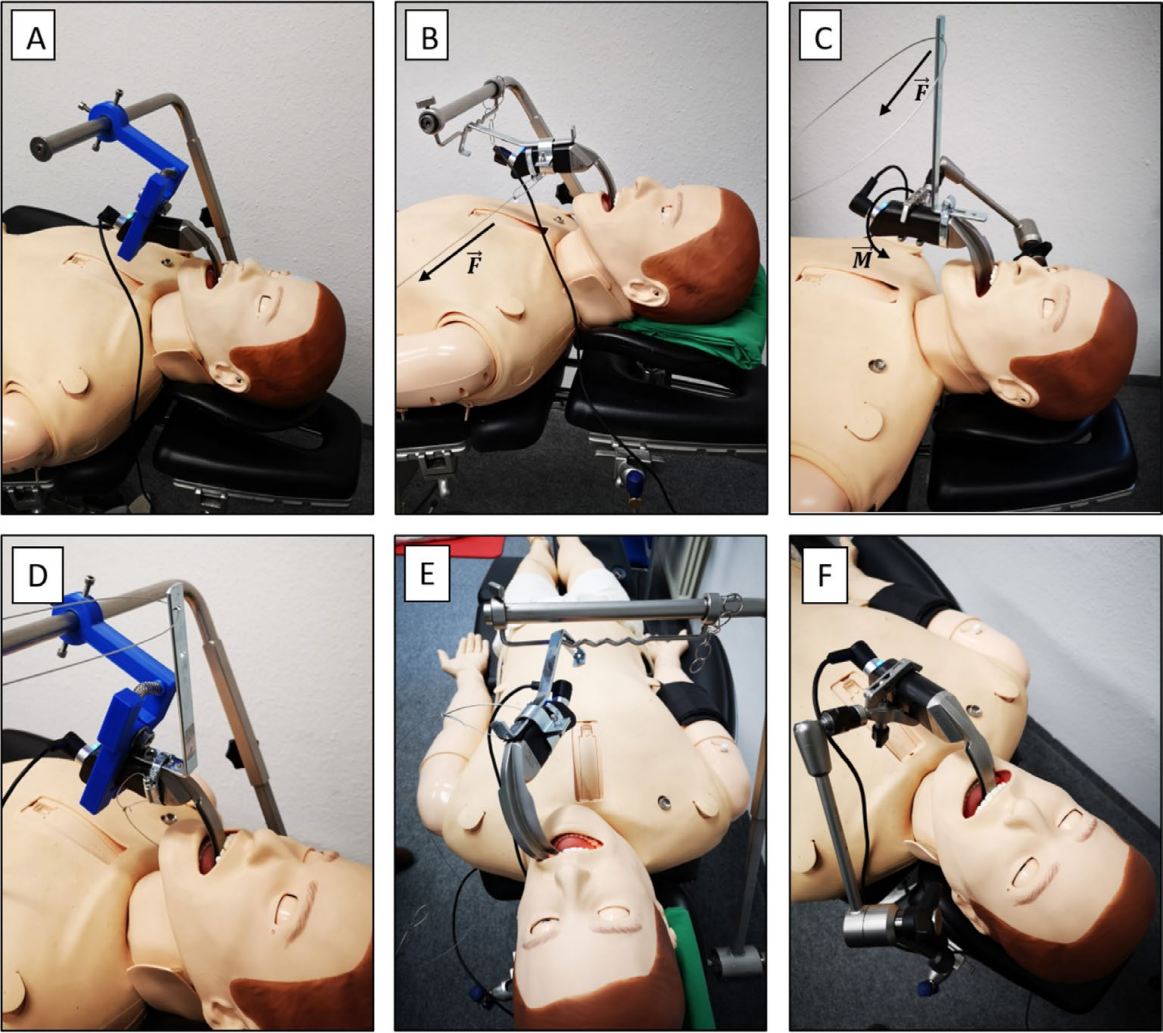
Experimental setup of the evaluated attachment methods for the video laryngoscope. (A + D) 3D‐printed clamp. (B + E) Metal bracket, with dislocation of the device shown in (E). The arrow labeled F indicates the direction of the applied force. (C + F) Articulated stand. The arrow labeled F indicates the direction of the applied force, while M represents the resulting torque.

For the experiment, an intubation and advanced cardiac life support dummy (Resusci Anne simulator, Laerdal, Stavanger, Norway) was positioned on a standard operating table. The video laryngoscope and the respective mounts were positioned to display all relevant laryngeal landmarks, which included the vocal cords, the vestibular folds, the anterior commissure, and the postcricoid region. External forces were applied to the surgical setup via thin steel cables, with tensile force gradually increased until the video laryngoscope lost visualization of one of the laryngeal structures, defining the trial endpoint. Forces were manually applied and continuously monitored, enabling similar force ramp‐up profiles across experimental trials. Figure 3 shows the experimental setup for each of the evaluated attachment methods.

Two scenarios simulating external forces applied to the surgical setup were investigated.

- In the first scenario, a lateral force perpendicular to the side of the handle of the video laryngoscope was applied.
- In the second scenario, a force was applied to an angle bracket attached to the laryngoscope handle, functioning as a lever. This generated torque around an axis longitudinal to the video laryngoscope grip.

For each attachment method, three trials were conducted for the first scenario. For the second scenario, five trials were conducted for each attachment method. The data analysis, evaluation, and graphical representation of the data was performed using MATLAB (Mathworks, Natick, USA). To compare the evaluated attachment methods regarding their resistance to the external forces, a one‐sided Wilcoxon rank sum test for independent samples was conducted. Statistical significance was considered for *p* < 0.05. Results are given in the form of median and 95% confidence interval (CI) for the position of the median unless stated otherwise.

## 3 Results

**FIGURE 4:**
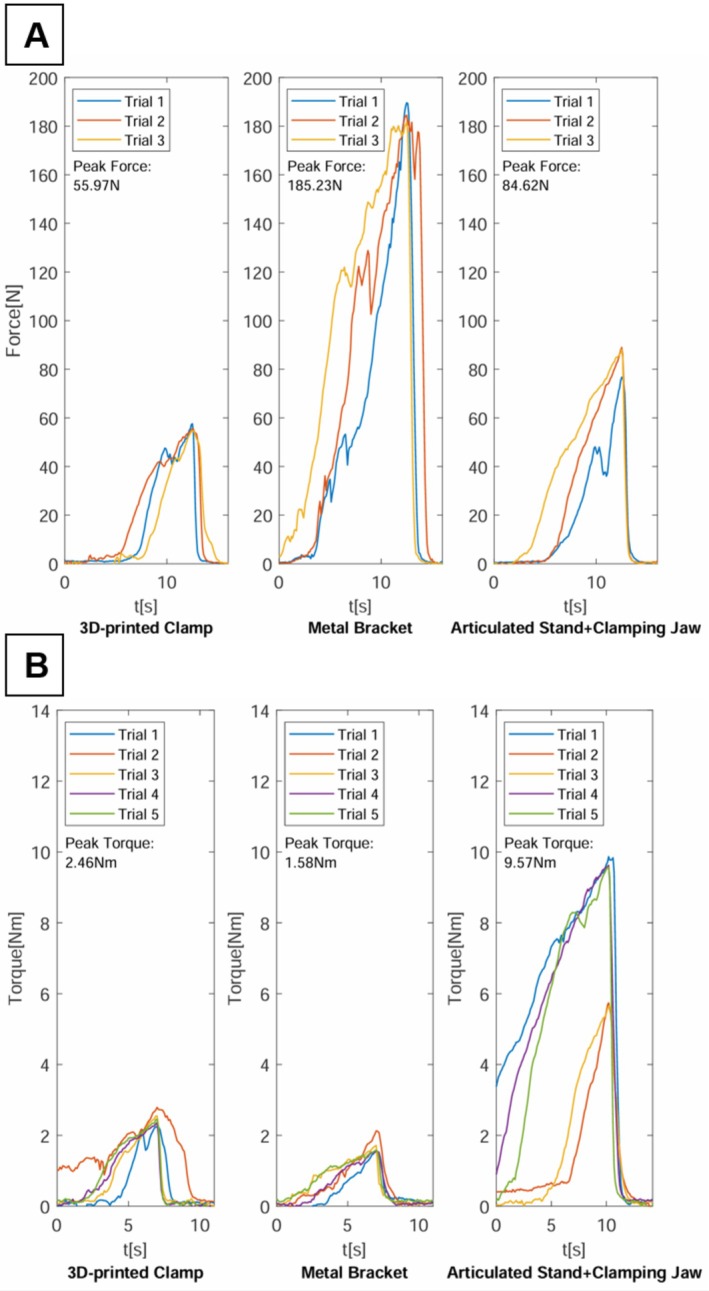
Forces and torques applied to the video laryngoscope in both scenarios and for each trial and attachment method. (A) First test scenario (lateral force). (B) Second test scenario (torque).

All three attachment methods facilitated successful visualization of the defined laryngeal landmarks using the video laryngoscope. The setup time for each method ranged from 2 to 5 min, which is considered acceptable for clinical use. Figure 4 illustrates the forces and torques measured during the experiments across both experimental scenarios and for each attachment method. Table 1 provides the median peak forces and torques that each attachment method could withstand before loss of visualization of the laryngeal landmarks across both scenarios.

**TABLE 1:** Median and 95% confidence interval for the position of the median of the peak forces in newton and torques in newton–meters resisted by each attachment method across all trials.

Mounting method	Peak Force Scenario 1 (N)		Peak Torque Scenario 2 (N m)	
	Median	95% CI	Median	(95% CI)
3D‐printed clamp	55.59	54.74–57.58	2.46	2.24–2.79
Metal bracket	184.49	181.59–189.61	1.58	1.57–2.13
Articulated stand + clamping jaw	88.61	76.73–88.98	9.57	5.65–9.87

The forces measured in the first scenario are depicted in Figure 4A. In this scenario, a force perpendicular to the lateral surface of the video laryngoscope handle was applied, as shown in Supporting Information 1 and 2. In this scenario, the metal bracket exhibited the greatest resistance to lateral forces, maintaining visualization of laryngeal landmarks up until a median force of 184.49 N (95% CI: 181.59–189.61 N) was applied. The articulated stand managed to withstand a median force of 88.16 N (95% CI: 76.73–88.98 N) before visualization was compromised. In contrast, the 3D‐printed clamp showed the lowest resistance against laterally acting forces, with interference of laryngeal visualization occurring at a median force of only 55.59 N (95% CI: 54.74–57.58 N). Statistical analysis confirmed that the force required to compromise visualization was significantly higher for the metal bracket compared to the articulated stand (184.49 N [95% CI: 181.59–189.61 N] vs. 88.16 N [95% CI: 76.73–88.98 N], *p* < 0.001) and the 3D‐printed clamp (184.49 N [95% CI: 181.59–189.61 N] vs. 55.59 N [95% CI: 54.74–57.58 N], *p* < 0.001).

Figure 4B illustrates the torque measured during the second scenario, where a torque was applied around the longitudinal axis of the laryngoscope handle, as shown in Supporting Information 3. The articulated stand demonstrated the greatest torque resistance, with a median torque of 9.57 N m (95% CI: 5.65–9.87 N m) resulting in the loss of visualization of laryngeal landmarks. The 3D‐printed clamp and the metal bracket showed substantially lower torque tolerance. The 3D‐printed clamp withstood a torque of 2.46 N m (95% CI: 2.24–2.79 N m), while the metal bracket exhibited a torque resistance of 1.58 N m (95% CI: 1.57–2.13 N m) before visualization of laryngeal structures was impaired. Statistical analysis revealed significantly higher torque resistance for the articulated stand compared to both the 3D‐printed clamp (9.57 N m [95% CI: 5.65–9.87 N m] vs. 2.46 N m [95% CI: 2.24–2.79 N m], *p* = 0.004) and metal bracket (9.57 N m [95% CI: 5.65–9.87 N m] vs. 1.58 N m [95% CI: 1.57–2.13 N m], *p* = 0.004).

## 4 Discussion

Previous studies that investigated the use of a curved video laryngoscope for surgical procedures in the head and neck area employed a two‐surgeon technique, where an assistant held the video laryngoscope steady while the primary surgeon performed the procedure [7, 8, 9, 10]. However, this two‐surgeon technique includes several disadvantages. The reliance on an assistant to hold the video laryngoscope may increase personnel costs and require close coordination between the assistant and primary surgeon to ensure optimal visualization. Additionally, the physical demand on the assistant to maintain the laryngoscope's stability throughout potentially lengthy procedures can lead to fatigue, compromising the consistency of the visual field.

In contrast, the attachment methods for the video laryngoscope presented in this study, similar to conventional suspension laryngoscopy, provide a stable surgical platform and static visualization of the operating field without requiring an additional assistant. This enables the procedure to be performed independently by a single surgeon.

During transoral surgery, considerable forces act on the operating laryngoscope [12]. Therefore, a reliable method of securing the video laryngoscope is essential to guarantee the safety of the patient. The metal bracket demonstrated remarkable resistance to lateral forces, attributable to the robust bolted connection between the video laryngoscope handle and S‐shaped connecting rail. However, its design limitation lies in the interface with the operating table crossbar, where the connecting rail lacks rigid fixation. This structural weakness resulted in significantly reduced rotational stability in the second scenario. A fixed connection between the metal bracket's connecting rail and crossbar, similar to the 3D‐printed clamp design, could enhance torque resistance.

The 3D‐printed clamp demonstrated significantly lower resistance against external forces compared to the alternative attachment methods across both testing scenarios. This inferior performance was primarily attributed to insufficient spring tension in the clamping mechanism, resulting in displacement of the video laryngoscope and compromised field of view under external forces. A higher‐tension spring mechanism or alternative methods to secure the laryngoscope handle, such as a buckle or screw‐type clamping system, could potentially improve the resilience of this attachment method against external forces.

The articulated stand demonstrated strong resistance to laterally applied forces in the first scenario and exhibited the best rotational stability among all evaluated attachment methods in the second scenario. This attachment method overall offers the best compromise between lateral force resistance and rotational stability. The excellent rotational stability can be attributed to the robust locking mechanism of the clamping jaw and the direct connection of the articulated stand to the operating table through the rotation socket.

According to a prior study by Schild et al. [12], intraoperative forces typically encountered during video laryngoscope‐guided surgery are approximately 50 N. In the first scenario of the present investigation, all evaluated attachment methods successfully resisted forces of at least 50 N without compromising laryngeal visualization. Based on these findings, we conclude that the tested attachment methods demonstrate sufficient robustness for practical application in a surgical setting. To date, no studies have investigated torque application during laryngoscopy using a curved video laryngoscope. Given that transoral laryngeal surgery is typically performed with the patient in the supine position under general anesthesia, the authors anticipate only minimal torque forces will be encountered. Based on the results of the present study, all three evaluated fixation methods are considered suitable for surgical use with respect to rotational stability.

A potential limitation of this study is the manual application of force. Alternatively, a motorized or ratchet‐based system could provide a more linear force ramp‐up profile which would have allowed for greater reproducibility. However, the chosen experimental setup was designed to reflect real‐world clinical conditions, where external forces acting on the laryngoscope are inherently more dynamic. To reproduce similar force ramp‐up profiles between different experimental trials, applied forces were continuously monitored.

A hysteresis effect was most noticeable in the 3D‐printed clamp setup, where the video laryngoscope completely disengaged from the clamping mechanism when external forces were applied. Consequently, upon force release, the visualization of laryngeal landmarks could not be recovered. A similar behavior was observed in the custom‐built metal bracket, due to the displacement of the bracket on the operating table crossbar. In contrast, the mechanical system of the articulated stand exhibited elastic behavior. When an external force was applied, a shift in the visual axis increased proportionally with the force. However, after releasing the external force, the articulated stand partially returned to its original position, with the view of the video laryngoscope centered again.

During the experiments, qualitative observations were made regarding the practicality and user‐friendliness of the different attachment methods. Given that endolaryngeal surgery usually is performed by a single surgeon, the positioning of the video laryngoscope should be achievable without the need for assistant support.

The main advantage of the 3D‐printed spring clamp is its ease of use. The spring mechanism enables quick and straightforward attachment and removal of the video laryngoscope. In contrast, the metal bracket secures the video laryngoscope by four screws, making adjustments or removal time‐consuming. Both the 3D‐printed clamp and the metal bracket are mounted to the operating table via a crossbar that uses a rotation socket for flexible alignment at various insertion angles. However, adjustments of the crossbar during the procedure typically require additional personnel as it is mounted to the siderail of the operating table. The articulated stand attaches easily to the operating table siderail via a rotation socket and allows for quick positional adjustments through its articulated joints. However, changing the position of the laryngoscope involves opening and resecuring the central mechanical clamp, which may also require an assistant's help.

Limitations of the study include the small sample size and the simplified testing conditions. In actual surgical settings, external forces are multidimensional, and force application is not limited to being strictly perpendicular to the handle or solely applying torque around a single axis. This complexity in real‐world force dynamics is not fully replicated in the experimental design, which may affect the generalizability of the results.

## 5 Conclusion

This study evaluated three attachment methods for a curved video laryngoscope in a surgical setting, assessing their resistance to external forces across two scenarios. The articulated stand emerged as the most effective attachment method, displaying good resistance to lateral forces in the first scenario and exceptional resistance to rotational forces in the second scenario. Additionally, the articulated stand was straightforward to handle, requiring only the fastening of a single central clamp to secure the entire setup in place. The metal bracket exhibited particularly good resistance against laterally acting forces, but its handling proved to be inflexible and more complex. The 3D‐printed clamp, although user‐friendly, demonstrated the lowest resistance to external forces in both testing scenarios. Future studies should investigate additional performance metrics, including setup efficiency and time to achieve optimal surgical field visualization. Additionally, clinical trials are needed to validate these findings in actual surgical procedures.

## Conflicts of Interest

The authors declare no conflicts of interest.

## Supporting information


**Supporting Information 1.** Video of the experimental procedure for the 3D printed clamp as attachment method, presenting the endolaryngeal and external view side by side in the Peak Force Scenario 1.

**Supporting Information 2.** Video of the experimental procedure for the metal bracket as attachment method, presenting the endolaryngeal and external view side by side in the Peak Force Scenario 1.

**Supporting Information 3.** Video of the experimental procedure for the articulated stand as attachment method, presenting the endolaryngeal and external view side by side in the Peak Torque Scenario 2.

## Data Availability

The CAD files for the 3D‐printed clamp as well as any oringinal data are available upon request from the authors and will be provided free of charge for scientific purposes.
